# Regular medication as a risk factor for intracranial aneurysms: A comparative case–control study

**DOI:** 10.1177/23969873221129080

**Published:** 2022-10-07

**Authors:** Ramazan Jabbarli, Marvin Darkwah Oppong, Mehdi Chihi, Thiemo Florin Dinger, Maryam Said, Jan Rodemerk, Philipp Dammann, Börge Schmidt, Cornelius Deuschl, Nika Guberina, Karsten H. Wrede, Ulrich Sure

**Affiliations:** 1 Department of Neurosurgery and Spine Surgery, University Hospital Essen, Essen, Germany; 2 Institute for Medical Informatics, Biometry and Epidemiology, University Hospital Essen, Essen, Germany; 3 Institute for Diagnostic and Interventional Radiology, Department of Neuroradiology, University Hospital Essen, Essen, Germany; 4 Department of Radiotherapy, University Hospital Essen, Essen, Germany

**Keywords:** intracranial aneurysm, subarachnoid hemorrhage, risk, medication, comorbidity

## Abstract

**Background:**

Previous medical history strongly contributes to the genesis of intracranial aneurysms (IA). A possible impact of regular medication on the occurrence of abdominal aortic aneurysms has been reported.

**Aim:**

To evaluate the value of regular medication on the risk of development and rupture of IA.

**Methods:**

Data on medication use and related comorbidities were obtained from the institutional IA registry. A 1:1 age- and sex-matched patient sample was collected from the population-based Heinz Nixdorf Recall Study with individuals from the same area.

**Results:**

In the analysis comparing IA cohort (*n* = 1960) with the matched normal population (*n* = 1960), the use of statins (adjusted odds ratio, 1.34 [95% confidence interval 1.02–1.78]), antidiabetics (1.46 [1.08–1.99]), and calcium channel blockers (1.49 [1.11–2.00]) was independently associated with higher risk of IA, whereas uricostatics (0.23 [0.14–0.38]), aspirin (0.23 [0.13–0.43]), beta-blockers (0.51 [0.40–0.66]), and angiotensin-converting enzyme inhibitors (0.38 [0.27–0.53]) were related to lower risk of IA. In the multivariable analysis within the IA cohort (*n* = 2446), SAH patients showed higher drug exposure with thiazide diuretics (2.11 [1.59–2.80]), but lower prevalence of remaining antihypertensive medication—beta-blockers (0.38 [0.30–0.48]), calcium channel blockers (0.63 [0.48–0.83]), angiotensin-converting enzyme inhibitors (0.56 [0.44–0.72]), and angiotensin-1 receptor blockers (0.33 [0.24–0.45]). Patients with ruptured IA were less likely to be treated with statins (0.62 [0.47–0.81]), thyroid hormones (0.62 [0.48–0.79]), and aspirin (0.55 [0.41–0.75]).

**Conclusions:**

Regular medication might impact the risks related to the development and rupture of IA. Further clinical trials are required to clarify the effect of regular medication on IA genesis.

## Introduction

Subarachnoid hemorrhage (SAH) resulting from rupture of an intracranial aneurysm (IA) presents a subtype of stroke with high case fatality and morbidity.^1^ Preventive measures by identifying individuals at risk for development and rupture of IA, and interventional treating of rupture-prone IA, are of paramount clinical importance.^1,2^

Besides non-modifiable risk factors (age, sex, certain systemic disorders,and genetic predispositions), there are also modifiable conditions influencing the risk of IA (arterial hypertension; use of tobacco, alcohol, and drugs).^3^ The second subgroup presents a potential therapeutic target for conservative management of individuals with IA.^4^ Along with the treatment of confounding comorbidities, the use of specific pharmacologic agents might also become a promising measure for the prevention of growth and rupture of IA. So far, the impact of aspirin and statins on the natural course of IA has been addressed in several experimental and clinical studies.^5–9^ Several studies have explored the possible effects of other drugs (particularly regular medication) on the formation of abdominal aortic aneurysms (AAA),^10–14^ but few have investigated this in relation to IA.^9,15,16^

In this observational case–control study, we compared the regular medication of a large consecutive IA cohort with an age- and sex-matched general population from the same geographic area. In addition, we analyzed the impact of regular medication on the rupture of IA.

## Materials and methods

### Patient population

This retrospective comparative study, which followed STROBE guidelines, analyzed two cohorts from the same time period and region (the German Ruhr area).

The IA cohort was based on the institutional observational study containing all consecutive cases with angiographically confirmed IA treated at the University Hospital Essen between January 2003 and June 2016 (*n* = 2446, aged 2 months – 90 years, 68.6% females). Any cases related to genetic/syndromal disorders, or resulting from trauma or infection, were excluded.

As the normal population (control cohort), we used data from the ongoing Heinz Nixdorf Recall (HNR; aged 45–74 years, 50.2% females) study and the Heinz Nixdorf MultiGenerationStudy (MGS; aged 18–90 years, 54.9% females), which assessed the prognostic value of modern risk stratification methods in a large unselected cohort of 4814 women and men (HNR; randomly selected between 2000 and 2003 from mandatory registries of residence of the cities Bochum, Essen, and Mülheim/Ruhr in the German Ruhr Area; response proportion at study baseline: 56%) as well as in their 2928 spouses and children (MGS; baseline examination between 2013 and 2016).^17^ Individuals in the HNR/MGS study underwent magnetic resonance imaging (MRI) of the brain to screen for any intracranial pathology. HNR study participants with IA identified during MRI screening or self-reported IA were excluded from the present analysis. Individual 1:1 matching by age and sex was applied to 5-year age groups from 18 years of age upwards. From each of the HNR/MGS families, only one individual was used for matching, to avoid any familial relationships within the control cohort. For 1960 IA cases a control could be randomly matched according to sex and age group, leaving 486 IA cases without a control mainly because of incompatible age (see Supplementary Table A1 (appendix p 3) for the comparison of characteristics of IA patients who were included in or excluded from the matched analysis).

This study complied with the requirements of the local Institutional Ethics Committee (Ethik-Kommission, Medizinische Fakultät der Universität Duisburg-Essen; registration number: 15-6331-BO) and was registered in the German clinical trial registry (DRKS; unique identifier: DRKS00008749). Written informed consent for patient data was provided by the patients or a legally authorized representative.

### Data management

As both studies were independently designed, the selection of variables for the present study was based on the congruency of data architecture in the databases. Accordingly, the data obtained from each database for further analysis included:

- the patient’s age and sex
- the presence of arterial hypertension and chronic cardiac diseases (at baseline)
- the regular medication: antihypertensive drugs according to five major classes (beta-blockers, calcium channel blockers, angiotensin-converting enzyme (ACE) inhibitors, angiotensin-1 receptor blockers, and thiazide diuretics), statins, thyroid medication (substitution hormones or thyrostatics), antidiabetics, uricostatics, and aspirin.

In addition, the occurrence of aneurysmal SAH was recorded in individuals from the IA cohort.

### Study endpoints and statistical analysis

For the association between drug exposure and IA presence, we generated a 1:1 age- and sex-matched analysis population from the baseline cohorts. Univariate and multivariable conditional logistic regression models were calculated to estimate the crude and adjusted odds for each drug group. The multivariable analysis was adjusted for the presence of drug-related cardiovascular comorbidities. For statins, antidiabetics, thyroid medication, and uricostatics, the underlying comorbidities were excluded from the analysis, as they were fully covered by the appropriate intake of these medications. Results were expressed as odds ratios (OR) or adjusted OR (aOR), as appropriate, with 95% confidence intervals (CI).

The association between the recorded drugs and SAH was analyzed in the IA cohort. Univariate correlations used Chi-square or Fisher’s exact tests, as appropriate. Multivariable binary logistic regression analysis—adjusted for the patients’ age, sex, and comorbidities—evaluated the independent association of the regular medication with IA rupture.

Statistical analyzes used PRISM (version 5.0; GraphPad Software Inc., San Diego, CA, USA) and SPSS (version 25; SPSS Inc., IBM, Chicago, IL, USA). Differences with a p-value < 0.05 were considered statistically significant.

## Results

To generate the individually 1:1 age- and sex-matched populations for the pooled analysis of risk factors for IA development, we selected 1960 participants from each cohort (*N* = 3920). Our analysis of the risk factors for IA rupture included data from the whole IA cohort (*n* = 2446; see Table 1 for the baseline characteristics). The study flowchart in figure A1 (appendix p 1) shows the selection process for both final cohorts.

**Table 1.** Baseline characteristics of IA cohort (*n* = 2446).

	Mean (±SD/range) or n (%)
**Demographic characteristics**	
Age, years	54.5 (±13.4)
Sex, female	1677 (68.6)
Ethnicity, non-Caucasian	125 (5.1)
**IA characteristics**	
Cases with SAH	1180 (48.2)
Cases with multiple IA	849 (34.7)
Number of IA/patient	1.5 (range, 1–9)
IA size, mm	8.3 (±5.8)
**Previous medical history: regular medication and comorbidities**	
Statins	347 (14.2)
Thyroid hormone replacement therapy	362 (14.8)
Thyrostatics	36 (1.2)
Antidiabetics	232 (9.5)
Uricostatics	56 (2.3)
Aspirin	69 (2.8)
Beta-blockers	593 (24.2)
Calcium channel antagonists	362 (14.8)
ACE inhibitors	531 (21.7)
Angiotensin-1 receptor blockers	264 (10.8)
Thiazide diuretics	291 (11.9)
Arterial hypertension	1577 (64.5)
Chronic cardiac diseases	380 (15.5)
Current smokers	586 (24.0)
Oncologic diseases	265 (10.8)
Musculoskeletal disorders	274 (11.2)
Peripheral arterial occlusive disease	369 (15.1)

ACE: angiotensin-converting enzyme; IA: intracranial aneurysm; SAH: subarachnoid haemorrhage; SD: standard deviation.

In the matched cohort, the mean age of the individuals with and without IA was similar: 55.4 years (±12.3). With 1342 females (68.5%) in each cohort, the sex ratio was also equal in the populations with and without IA. Among matched IA cases, 937 individuals (47.8%) suffered aneurysmal SAH.

**Figure 1.**
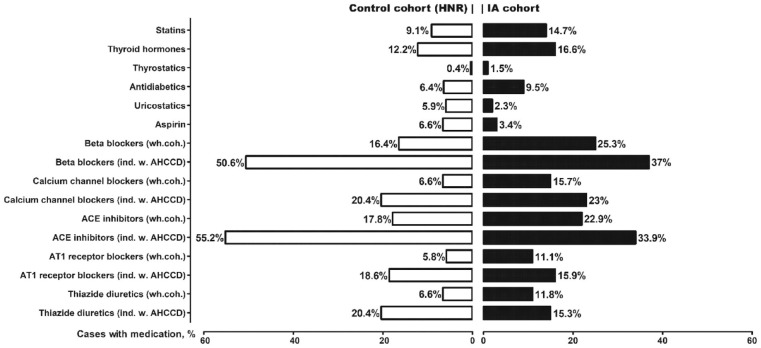
Prevalence of regular medication (in %) in the age- and sex-matched IA and control (HNR) cohorts. The rate of antihypertensive drugs is given for the whole cohort (“wh.coh.”) and separately for individuals with arterial hypertension or chronic cardiac diseases (“ind. W. AHCCD”). ACE: angiotensin-converting enzyme; AT1: angiotensin I; HNR: Heinz Nixdorf Recall Study; IA: intracranial aneurysm.

Figure 1 shows the prevalence of regular medication in both cohorts. We measured the prevalence of antihypertensive medication separately for individuals with known arterial hypertension or chronic cardiac diseases. Figure A2 (appendix p 2) shows the results of the univariate analysis with crude OR for each medicine.

**Figure 2.**
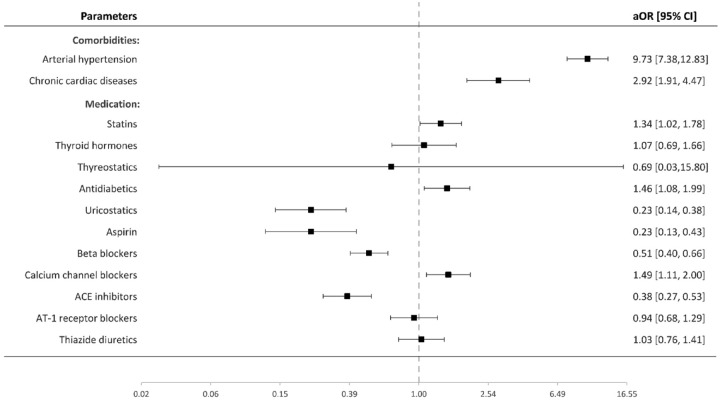
Value of regular medication and included co-morbidities in the development of IA: multivariable conditional regression analysis. ACE: angiotensin-converting enzyme; AT1: angiotensin I; IA: intracranial aneurysm.

In the final multivariable analysis (Figure 2), use of statins (aOR 1.34 [95% CI 1.02–1.78], *p* = 0.039), antidiabetics (1.46 [1.08–1.99], *p* = 0.015), and the calcium channel blockers (1.49 [1.11–2.00], *p* = 0.008) was independently associated with a higher risk of IA, whereas medication with uricostatics (0.23 [0.14–0.38], *p* < 0.0001), aspirin (0.23 [0.13–0.43], *p* < 0.0001), beta-blockers (0.51 [0.40–0.66], *p* < 0.0001), and ACE inhibitors (0.38 [0.27–0.53], *p* < 0.0001) was related to lower risk of IA. Presence of arterial hypertension (9.73 [95% CI 7.38–12.83], *p* < 0.0001) or chronic cardiac diseases (2.92 [1.91–4.47], *p* < 0.0001) was associated with the risk of IA. The remaining groups of regular medication showed no significant associations with the presence of IA.

**Figure 3.**
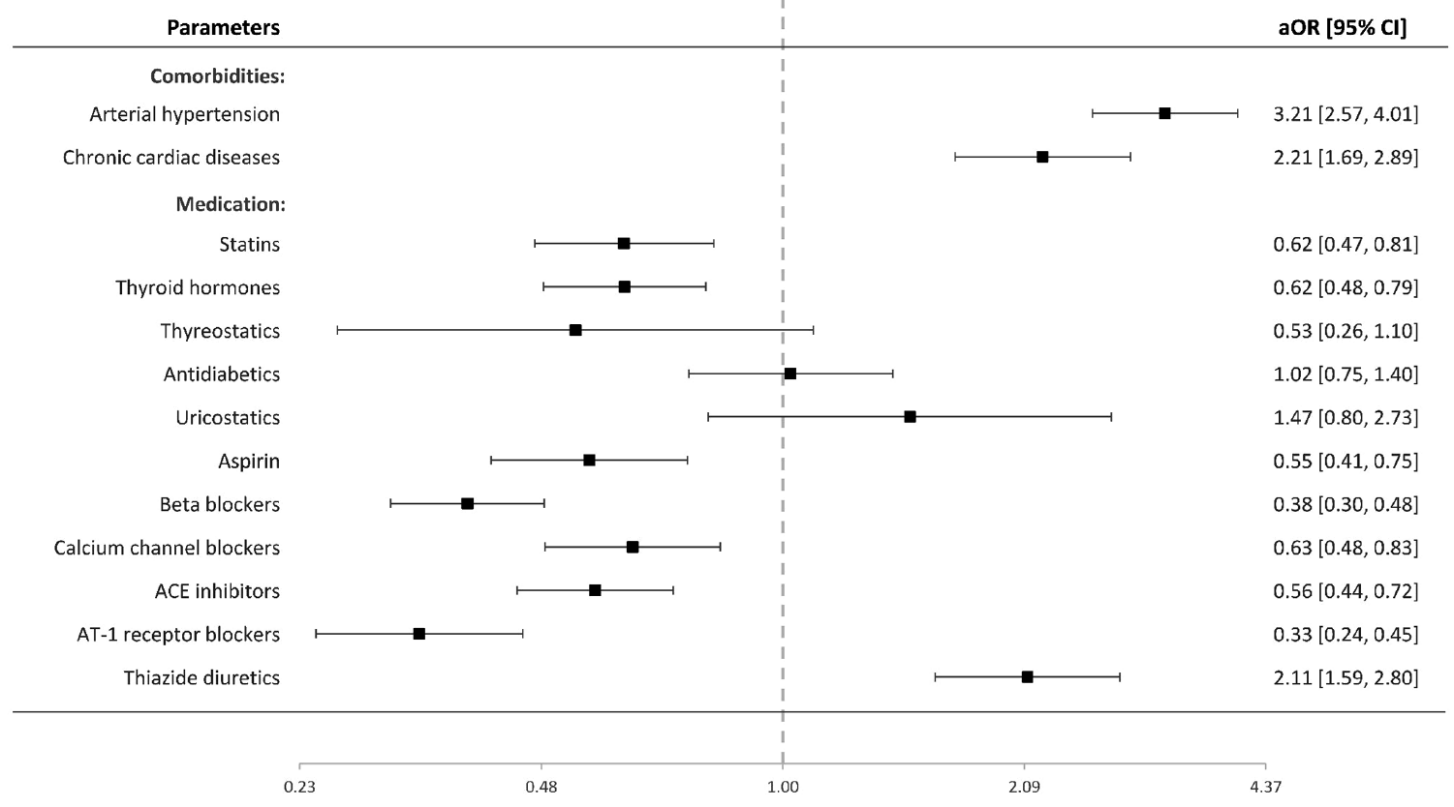
Value of regular medication and included co-morbidities for IA rupture: multivariable binary logistic regression analysis. ACE: angiotensin-converting enzyme; AT1: angiotensin I; IA: intracranial aneurysm.

**Figure 4.**
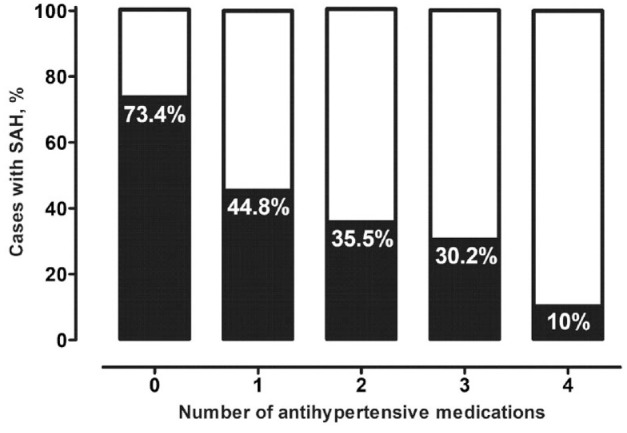
Association between the number of SAH-protective antihypertensive medication classes (beta-blockers, calcium channel antagonists, ACE inhibitors, and angiotensin-1 receptor blockers) and occurrence of SAH in individuals with IA and known arterial hypertension, and/or chronic cardiac diseases. ACE: angiotensin-converting enzyme; IA: intracranial aneurysm; SAH: subarachnoid hemorrhage.

In the IA cohort, 1180 patients (48.2%) experienced SAH following IA rupture. Figure A3 (appendix p 2) presents crude ORs for the effect of regular medication on SAH risk. In the multivariable analysis (Figure 3), SAH patients showed higher exposure with diuretics (aOR 2.11 [95% CI 1.59–2.80], *p* < 0.0001), but lower with the remaining antihypertensive drugs—beta-blockers (0.38 [0.30–0.48], *p* < 0.0001), calcium channel blockers (0.63 [0.48–0.83], *p* = 0.001), ACE inhibitors (0.56 [0.44–0.72], *p* < 0.0001), and angiotensin-1 receptor blockers (0.33 [0.24–0.45], *p* < 0.0001). Figure 4 shows the cumulative effect of beta-blockers, calcium channel blockers, ACE inhibitors, and angiotensin-1 receptor blockers on the risk of IA rupture among individuals with arterial hypertension, and/or chronic cardiac diseases.

With regard to the remaining regular medication, SAH patients were less likely to be treated with statins (aOR 0.62 [95% CI 0.47–0.81], *p* = 0.001), thyroid hormones (0.62 [0.48–0.79], *p* < 0.0001), and aspirin (0.55 [0.41–0.75], *p* < 0.0001), but showed no correlation with antidiabetics (1.02 [0.75–1.40], *p* = .883), uricostatics (1.47 [0.80–2.73], *p* = .217), and thyrostatics (.53 [0.26–1.10], *p* = .087). Finally, patients’ age (aOR: 0.99 per year age increase [95% CI 0.98–0.99], *p* < 0.0001), arterial hypertension (3.21 [2.57–4.01], *p* < 0.0001), and chronic cardiac diseases (2.21 [1.69–2.89], *p* < 0.0001) were associated with the risk of SAH.

## Discussion

In this comparative case–control study, we examined the effects of regular medication on the prevalence of IA and SAH. There are substantial differences in the drug patterns between the age- and sex-matched individuals with and without IA, as well as among IA carriers with and without IA rupture. Our results suggest a possible impact of regular medication on the natural course of IA.

Recent imaging and histopathological studies addressing the pathophysiology of IA formation in the vessel wall support the hypothesis that chronic vascular injury arises from long-lasting hemodynamic stress, with subsequent gradual degeneration and reorganization of the affected arterial wall (mostly on bifurcations as predilection sites)—a process perpetuated by various inflammatory and autoimmune pathways, intraluminal thrombosis, and oxidative stress.^18,19^

To date, conservative management of patients with IA is limited to recommending proper control of modifiable risk factors such as arterial hypertension or smoking.^3^ Although there is some evidence to support the clinical use of various drugs to affect the pathophysiological processes in the IA wall,^7^ there is currently no accepted medical treatment for IA. In this context, the possible impact of regular medication on the risk of development and rupture of IA is crucial: it could help inform more accurate risk assessment and treatment selection. However, clinical evidence is sparse for the interactions between different drug classes and IA formation.^5,8,9,15,16,20–23^

The results of the present study suggest that IA risk could be open to pharmaceutical manipulation. However, when interpreting our findings, limitations in the study design should be carefully considered. In particular, associations between the use of certain regular treatments and IA risk might reflect the impact of the underlying disease rather than the direct action of a drug. This applies particularly to the observed correlation between higher risk of IA and the use of statins or antidiabetics. Moreover, the importance of medication adherence for treatment outcome should also be considered when interpreting our study results. So, in a recent large survey among individuals with chronic diseases, 62% forgot to take medications and 37% had run out of their medication within a year.^24^ Accordingly, the compliance of patients with regard to regular medication intake also might have a substantial impact on the observed associations in the present study.

The role of diabetes mellitus in IA genesis is controversial. Although higher risk of IA growth in individuals with diabetes has been reported,^25^ several studies have shown a lower risk of IA formation and/or rupture in patients using glucose-lowering medications.^8,21^ While its association with vasculopathy is clear, little is known about how diabetes could affect the formation of IA.

Diabetes and dyslipidemia are considered the key factors for the development of atherosclerosis, a chronic inflammatory vascular disease.^26^ There are many similarities in the genesis of atherosclerosis and aneurysms in the vessel wall, since the inflammatory processes responsible for the progression of atherosclerosis largely overlap with those underlying IA formation.^27^ Furthermore, atherosclerotic changes in the IA wall seem to characterize unruptured, rather than ruptured, IA.^18^ This may explain why a previous study showed a lower rate of statins use in SAH individuals than in patients with unruptured IA.^9^ Thus, our findings of a higher rate of statins use in IA individuals (*vs* non-IA cohort) on the one hand, and a lower rate of statins in patients with SAH (*vs* unruptured IA cases) on the other, align with the current clinical evidence. Although dyslipidemia promotes the inflammatory processes leading to IA formation, it also seems to contribute to IA wall stabilization, which protects against IA rupture. Further research is needed to clarify the complex interaction of lipid disorders with IA formation.

Somewhat surprising was the impact of uricostatics on the risk of IA development. Uricostatics are typically xanthine oxidase inhibitors given to treat hyperuricemia. By activating inflammatory pathways, hyperuricemia was reported to increase the risk of atherosclerosis and other cardiovascular diseases.^26^ In contrast, IA carriers were less frequently treated with uricostatics in our comparative study. This effect might be related to the antioxidative pharmacologic effect of uricostatics. In addition, uric acid (as the metabolic end product of purine synthesis) also has some antioxidative properties.^26^ Therefore, the protective effect of uricostatics (or, in part, of hyperuricemia) might be related to the suppression of oxidative stress in the injured intracranial vasculature responsible for IA development.^18^

One of the most important findings of our study was the effect of aspirin on the risk of development and rupture of IA. As inflammation and intraluminal thrombosis are considered crucial in the pathogenesis and growth of IA,^3^ aspirin—as a widely used pharmacologic agent affecting both pathways—has been intensively analyzed with regard to its potential benefits and harms for patients with IA. Despite several positive reports, a recent meta-analysis failed to show any protective effect of aspirin on the mid- and long-term risk of SAH.5 Our data show for the first time the protective effect of aspirin on the risks of IA formation and rupture. More robust evidence is expected from the currently ongoing prospective randomized open-label multicentric trial (PROTECT-U) assessing the effect of aspirin on the risk of growth and rupture of IA.^28^

Of particular interest are our results evaluating various antihypertensive drugs. To date, only two studies^15,23^ have addressed the risk for IA development with antihypertensive medication; neither assessed drug classes separately, and the results were not significant. In our study, the use of ACE inhibitors was associated with a lower risk of IA—perhaps explained by activation of the renin–angiotensin system.^12^ Previous clinical trials on AAA showed that ACE inhibitors can inhibit their growth and rupture, probably by reducing vascular inflammation, augmenting systemic collagen synthesis, and reducing the stiffness of the aortic wall.^10–12^ In our analysis, beta-blockers were also related to lower IA risk. Previous human and animal studies have demonstrated a protective effect of beta-blockers on the risk of AAA growth and rupture^13^—perhaps linked to their inhibition of cardiac contractility and wall tension, as well as their antihypertensive activity.^13,14^ In contrast to ACE inhibitors and beta-blockers, calcium channel blockers seem to increase the risk of IA development, as also reported for AAA.^11^ The aneurysm-triggering effect of calcium channel blockers might be mediated by the potentiation of elastase and subsequent increase of vascular stiffness^11^; this could lead to increased wall shear stress, and thus a higher risk of IA developing.^29^

Regarding the risk of IA rupture and blood pressure medication, Yoshimura et al.^9^ reported lower risk of SAH with antihypertensive drugs; another study found a higher risk of SAH in patients with irregular use of antihypertensive medication.^16^ Our results support the protective effect of four major antihypertensive drug classes (beta-blockers, ACE inhibitors, calcium channel antagonists, and angiotensin-1 receptor blockers) on the risk of IA rupture, highlighting the importance of proper blood pressure treatment in individuals with IA.

In contrast, the use of thiazide diuretics was associated with a higher risk of SAH in our comparative study. There are currently no related clinical or experimental data to shed light on this association. Thiazide diuretics are recommended as first-line drugs for initial therapy of hypertension, but can cause different metabolic complications like alkalosis, hyperuricemia, decreased urinary calcium excretion, glucose intolerance, and lipid abnormalities.^30^ In turn, these complications, when untreated, might trigger IA progression. When taking into account the role of thiazide diuretics in treatment of arterial hypertension, there is an urgent need for further experimental and clinical studies aiming to confirm our findings and to elucidate the eventual pathophysiologic background of the impact of thiazide diuretics on the rupture risk of IA.

Finally, IA patients with thyroid hormone replacement therapy were less likely to present with SAH. There are small series reporting a higher prevalence of atherosclerosis^31^ and unruptured IA^32^ in individuals with hypothyroidism. Therefore, hypothyroidism and/or thyroid hormone replacement therapy might promote the processes in the intracranial vasculature contributing to stabilization of the IA wall and protection from rupture. Further research is warranted to explore the pathophysiological mechanisms to explain the observed associations between SAH risk and treatment with thiazide diuretics or thyroid hormones.

As discussed earlier, the major limitation of the present study concerns our inability to adjust the results for the presence, onset, and duration of all relevant underlying morbid conditions; the absence of data on timing of drug initiation and changes within/across the drug classes; and a lack of detailed information on prescribed dosages and patient adherence. Despite adjustment for some but not all IA-relevant factors (e.g. tobacco and alcohol consumption), residual confounding should be considered when interpreting our results, as epidemiological associations alone do not represent causal relationships.^12^ Finally, single-center design of the study should also be mentioned, necessitating further confirmation of our findings in external IA cohorts.

## Conclusions

Regular medication might impact the risks related to the development and rupture of IA. However, the role of duration of underlying diseases and the timing of initiating appropriate medication treatment requires further clarification. Large (multi-)national registry-based or prospective clinical trials are warranted to explore the effect of regular medication on the development of IA.

## Supplemental Material

sj-docx-1-eso-10.1177_23969873221129080 – Supplemental material for Regular medication as a risk factor for intracranial aneurysms: A comparative case–control study Click here for additional data file. Supplemental material, sj-docx-1-eso-10.1177_23969873221129080 for Regular medication as a risk factor for intracranial aneurysms: A comparative case–control study by Ramazan Jabbarli, Marvin Darkwah Oppong, Mehdi Chihi, Thiemo Florin Dinger, Maryam Said, Jan Rodemerk, Philipp Dammann, Börge Schmidt, Cornelius Deuschl, Nika Guberina, Karsten H. Wrede and Ulrich Sure in European Stroke Journal
