# Supplementary material for: Regular medication as a risk factor for intracranial aneurysms: A comparative case–control study
Source: Eur Stroke J. 2022 Oct 7;8(1):251–8. doi: 10.1177/23969873221129080 (PMC10069188; doi:10.1177/23969873221129080)
Supplement: sj-docx-1-eso-10.1177_23969873221129080 – Supplemental material for Regular medication as a risk factor for intracranial aneurysms: A comparative case–control study [file sj-docx-1-eso-10.1177_23969873221129080.docx]

SUPPLEMENTARY MATERIAL

Regular medication as a risk factor for intracranial aneurysms: a comparative case-control study

Ramazan Jabbarli^1^^[[1]](#footnote-1)^*, MD; Marvin Darkwah Oppong^1^, MD; Mehdi Chihi^1^, MD; Thiemo Florin Dinger^1^, MD; Maryam Said^1^, MD; Jan Rodemerk^1^; Philipp Dammann^1^, MD; Börge Schmidt^2^, PhD; Cornelius Deuschl^3^, MD; Nika Guberina^4^, MD; Karsten H. Wrede^1^, MD; Ulrich Sure^1^, MD

^1^Department of Neurosurgery and Spine Surgery, University Hospital Essen, Germany
^2^Institute for Medical Informatics, Biometry and Epidemiology, University Hospital Essen, Essen, Germany
^3^Institute for Diagnostic and Interventional Radiology, Department of Neuroradiology, University Hospital Essen, Germany
^4^Department of Radiotherapy, University Hospital Essen, Germany

# *Figure A1*: Study design


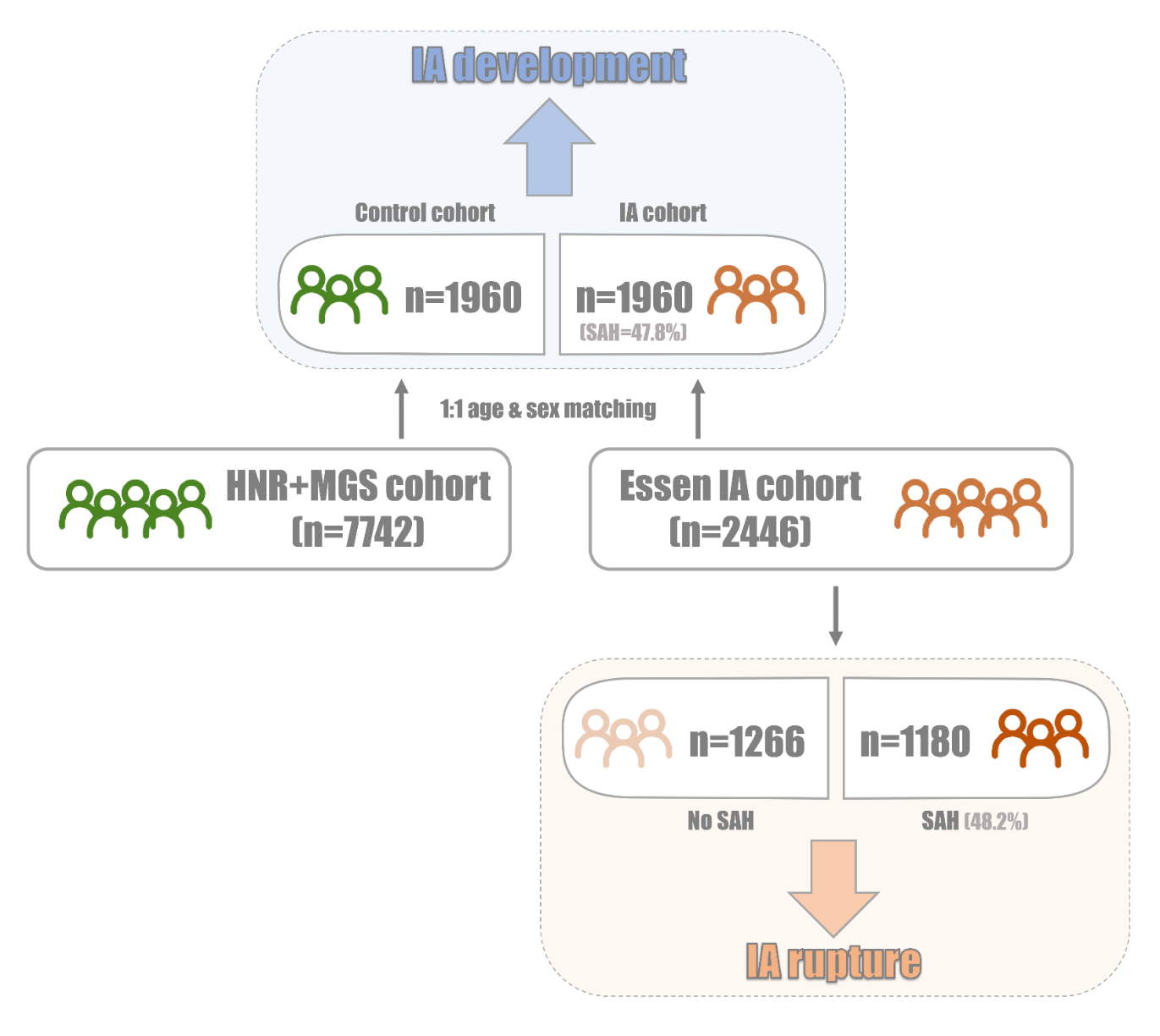


# *Figure A2*: Effect of drug exposure on the risk of IA presence: univariate conditional regression analysis


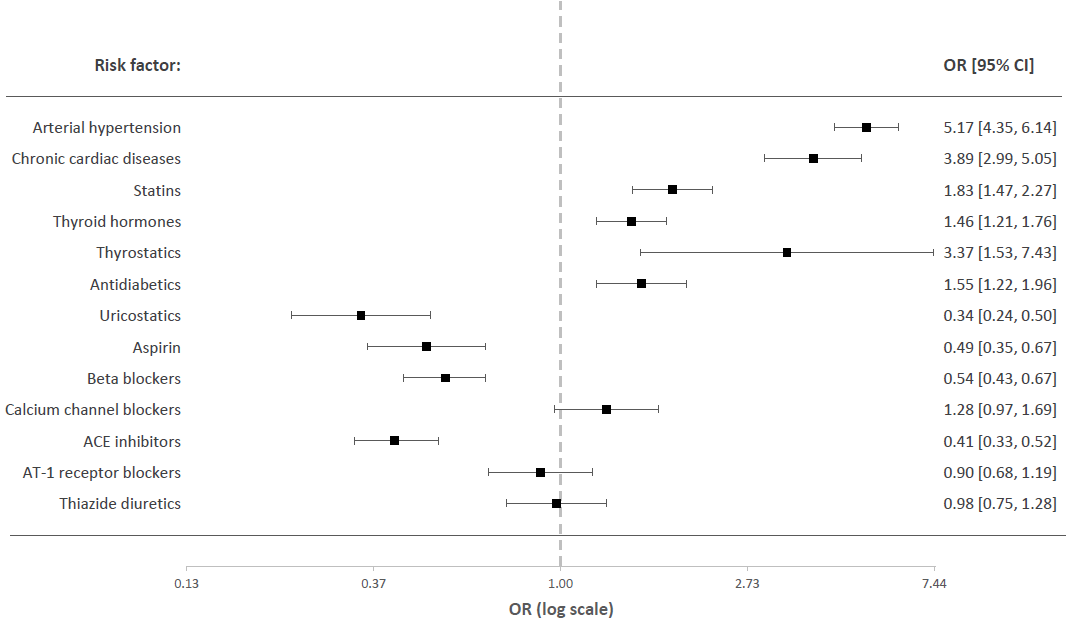


# *Figure A3*: Effect of drug exposure on the risk of SAH in the IA cohort: univariate analysis


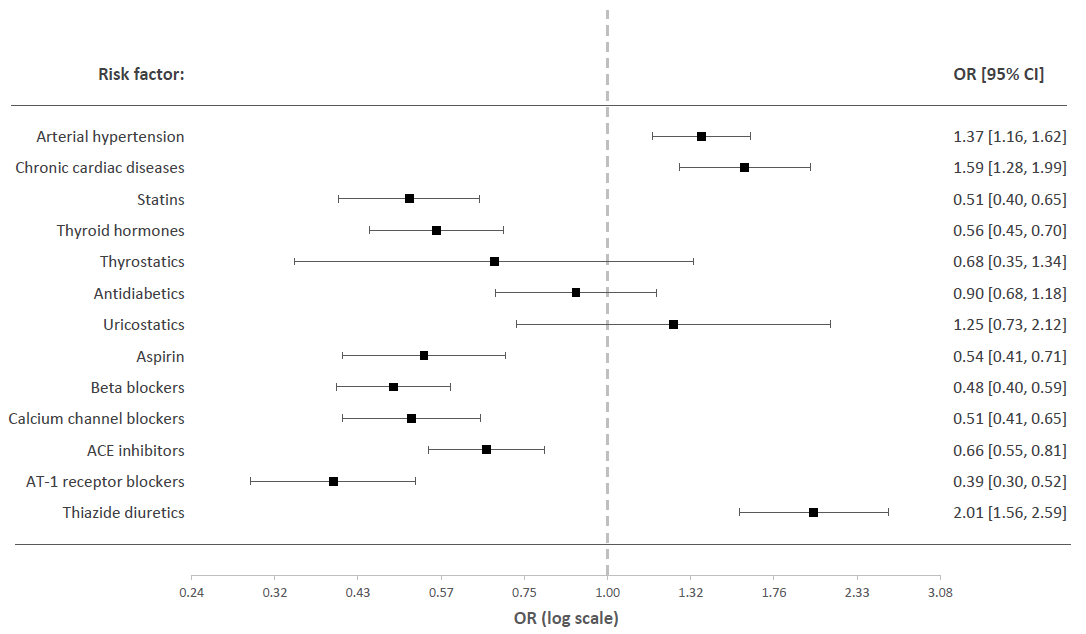


# *Table A1*: Univariate analysis comparing the baseline characteristics and medical history of patients in the IA cohort excluded from vs. included in the matched analysis

|  | **Mean (±SD/range) or OR (95% CI)** | **p-value** |
| --- | --- | --- |
| **Demographic characteristics** | |  |
| Age, years | 50.9 (±16.6) vs. 55.4 (±12.3) | **<0.0001** |
| Sex, female | 1.02 (0.83 – 1.27) | 0.829 |
| Ethnicity, non-Caucasian | 0.75 (0.49 – 1.14) | 0.207 |
| **IA characteristics** | |  |
| Cases with SAH | 0.93 (0.77 – 1.14) | 0.512 |
| Cases with multiple IA | 1.06 (.86 – 1.30) | 0.634 |
| Number of IA/patient | 1.5 (±0.9) vs. 1.5 (±0.9) | 0.709 |
| IA size (the largest one), mm | 8.0 (±5.6) vs. 8.4 (±5.8) | 0.130 |
| **Regular medication and related comorbidities** | |  |
| Statins | 1.10 (0.82 – 1.47) | 0.564 |
| Thyroid hormone replacement therapy | 1.55 (1.15 – 2.10) | **0.003** |
| Thyrostatics | 1.57 (0.61 – 4.06) | 0.410 |
| Antidiabetics | 1.02 (0.73 – 1.43) | 1.000 |
| Uricostatics | 1.03 (0.53 – 2.01) | 1.000 |
| Aspirin | 1.09 (0.59 – 2.00) | 0.880 |
| Beta-blockers | 1.18 (0.93 – 1.49) | 0.196 |
| Calcium channel antagonists | 1.28 (0.95 – 1.71) | 0.119 |
| ACE inhibitors | 1.26 (0.98 – 1.62) | 0.076 |
| Angiotensin-1 receptor blockers | 1.07 (0.78 – 1.48) | 0.746 |
| Thiazide diuretics | 0.99 (0.73 – 1.34) | 0.938 |
| Arterial hypertension | 1.23 (1.00 – 1.51) | 0.052 |
| Chronic cardiac diseases | 1.24 (0.93 – 1.65) | 0.164 |

Abbreviations: SD: standard deviation; OR: odds ratio; SAH: subarachnoid hemorrhage; IA: intracranial aneurysm.

1. * Corresponding author at [ramazan.jabbarli@uk-essen.de](mailto:ramazan.jabbarli@uk-essen.de) [↑](#footnote-ref-1)
